# Xenotransplantation of Porcine-Derived Products: Attitudes of Muslims and Jews Based on Religion, Culture, Ethical Views, and Perceived Knowledge

**DOI:** 10.3390/healthcare14213614

**Published:** 2026-10-26

**Authors:** Mahdi Tarabeih, Dorit Weil Lotan, Wasef Na’amnih

**Affiliations:** 1 School of Nursing Sciences, The Academic College of Tel-Aviv-Yaffo, 2 Rabenu Yeruham St, Tel Aviv 6818211, Israel; mahdita@mta.ac.il (M.T.); doritw@mta.ac.il (D.W.L.); 2 Department of Epidemiology and Preventive Medicine, School of Public Health, Gray Faculty of Medicine, Tel Aviv University, Tel Aviv 6997801, Israel

**Keywords:** Xenotransplantation, Porcine-derived materials, religious beliefs, cultural competence, informed consent, Islam, Judaism

## Abstract

**Highlights:**

**What are the main findings?**

• Both Muslim and Jewish participants showed limited religious knowledge about porcine xenotransplantation. Across the six organ-specific knowledge items, 15.08% of Muslim participants and 9.83% of Jewish participants selected the available “I don’t know” option. The composite Opinion difference was small and not statistically significant (*p* = 0.092).
• Knowledge and Opinion were strongly associated in both groups. Demographic associations varied between groups; the unconstrained mediation path model was saturated, and the formal comparison rejected equality of all 13 structural coefficients.

**What are the implications of the main findings?**

• Culturally sensitive, religion-specific informed consent and patient education strategies are needed rather than a uniform approach, since the drivers of acceptance differ substantially between Muslim and Jewish patients.
• Closing religious knowledge gaps requires coordinated efforts between healthcare providers and religious authorities, particularly for Muslim patients, for whom factual/biomedical information alone appears insufficient to shift attitudes.

**Abstract:**

**Background/Objectives:** Xenotransplantation with genetically modified pig organs offers a potential response to the global shortage of donor organs. Although both Islam and Judaism may permit exceptions when treatment is medically necessary, public awareness of these rulings remains limited. This study examined knowledge and attitudes among Muslim and Jewish adults regarding the medical use of porcine-derived organs. **Methods:** A cross-sectional study was conducted among 884 Muslims and 916 Jews aged 18–81 years. Participants completed an anonymous online questionnaire distributed through convenience and snowball sampling. Six organ-specific items assessed perceived religious knowledge using six ordered permissibility categories and a separate “I don’t know” option, and attitudes using a 7-point Likert scale. **Results:** Across the six knowledge items, 15.08% of Muslim responses and 9.83% of Jewish responses were “I don’t know.” Mean composite Knowledge scores were 2.69 and 2.49, respectively (*p* = 0.025; d = −0.113, Jewish minus Muslim). Composite Opinion means were 3.04 and 2.87 (*p* = 0.092; d = −0.080). The mediation model included 772 Muslims and 846 Jews; adjusted Knowledge–Opinion coefficients were β = 0.95 and 0.79. The free model was saturated (df = 0); constraining all 13 structural coefficients to equality across groups worsened fit (Δχ^2^(13) = 2718.19, *p* < 0.001). **Conclusions:** Public awareness of religious considerations surrounding porcine-derived transplantation was limited. The findings support culturally sensitive education and informed-consent processes while respecting individual autonomy and variation within religious communities. Strong Knowledge–Opinion associations require caution about common-method effects and do not establish causal mediation or actual treatment choices.

## 1. Introduction

Xenotransplantation (XTx) of organs from pigs is considered ritually impure in both Islam and Judaism [1,2]. Clinical trials involving porcine organs have advanced significantly in recent years [3]. Previous studies have shown that religious, cultural, and educational factors play a decisive role in shaping acceptance of xenotransplantation, even among transplant patients themselves [4,5,6,7]. Such findings underscore the importance of understanding religious and cultural perspectives when developing patient-centered approaches.

Porcine organs offer practical advantages for transplantation due to their availability, anatomical compatibility, and lower public objection compared to those from primates [8,9,10]. Notable advances include genetically engineered pig heart transplants [11,12,13,14] and the transplantation of a pig kidney into a brain-dead patient [14,15,16,17]. Despite these achievements, socio-cultural and ethical barriers remain central challenges [18,19,20,21]. Since informed consent is a crucial ethical consideration, ethicists argue that withholding information about animal-derived medical products undermines patient autonomy [22,23,24].

Islamic law explicitly prohibits the consumption of pork (Holy Quran 5:3, 6:145), but also upholds the principle that “necessities may supersede prohibitions” [25,26,27]. This concept allows exceptions in life-threatening cases, with rulings emphasizing consultation with religious leaders [25,26]. Similarly, Jewish law forbids pork consumption (Leviticus 11:7–8) but prioritizes the commandment of pikuach nefesh, preservation of human life [28,29,30]. Both traditions recognize that saving life overrides dietary restrictions [31,32,33,34].

Muslim jurisprudence applies Darūrah, permitting porcine use in cases of necessity, though fatwas restrict such use to emergencies [26,31,32,33]. In Jewish law, rabbinic rulings since the 1500s have sanctioned animal use for medical purposes, including porcine insulin and heart valves, under the principle that saving life takes precedence [34,35,36,37,38,39,40,41,42].

Previous studies show variability in acceptance between the various faith groups, but religious leaders chiefly agree that porcine-derived products may be used when no alternatives exist [43,44,45,46,47]. Nonetheless, patients may experience distress when recommended treatments involve porcine products, reflecting both religious and psychological concerns [40,48,49,50,51,52,53].

This study’s primary contribution is its integration of theological discourse with empirical analysis, moving beyond prior descriptive work on religious views toward transplantation. This approach clarifies how core principles within Islam and Judaism inform attitudes toward medical practices, particularly the use of porcine-derived organs, while recognizing variations between liberal and conservative interpretations [46,47]. Few studies have specifically examined how religious beliefs shape attitudes toward XTx [45,54,55,56]. Several studies shows that Faith often functions as a coping mechanism for patients dealing with an illness [33,57,58,59], leading some individuals to avoid porcine-based treatments unless transformed through processes such as Istihala [32,46,60]. Pork is explicitly prohibited in Jewish dietary law, as stated in Leviticus 11 and Deuteronomy 14. Similarly, the Qur’an contains multiple verses prohibiting the consumption of pork (e.g., Surat al-Baqarah 2:172–173). However, it also offers a critique of certain Jewish dietary restrictions that extend beyond the original prohibitions (Surah An-Nahl 16:115–118).

Given that pigs are regarded as ritually impure in Islam and Judaism, Muslim and Jewish patients may be hesitant to receive porcine-derived organ transplants [15,60,61]. There is also a potential for psychological distress when individuals receive animal-derived treatments without their knowledge [29]. Religious sensitivities and their psychological implications underscore the need for empirical, comparative research on how theological beliefs shape health attitudes. Our study aimed to explore the knowledge and attitudes of Muslim and Jewish communities towards the medical use of porcine-derived organs, to evaluate their perspectives on the religious appropriateness of pigs for human XTx, and to examine differences between the two groups. The study addressed the following five research questions:

(1) What do Muslims and Jews know about the religious permissibility of using porcine-derived organs for medical purposes?
(2) What are the opinions of Muslims and Jews regarding the permissibility of using organs of porcine origin for medical purposes?
(3) Is knowledge that porcine-derived organ use would be religiously permissible associated with a more favorable attitude toward it, and conversely, is perceiving it as prohibited associated with a less favorable attitude?
(4) Do Muslims and Jews differ in their knowledge about the permissibility of using porcine-derived organs for medical purposes?
(5) Do Muslims and Jews differ in their opinions regarding the permissibility of using porcine-derived organs for medical purposes?

## 2. Materials and Methods

### 2.1. The Study Design and Population

A cross-sectional study was conducted among Muslim and Jewish adults residing in Israel. Participants were recruited from the Northern, Central, and Southern regions of the country. Eligibility criteria included age ≥ 18 years, self-identification as Muslim or Jewish, residence in Israel, and provision of informed consent. Prior personal experience with organ transplantation, including experience among family members or other close associates, was not assessed and was not an inclusion or exclusion criterion for sample selection. Individuals who did not complete the questionnaire in full were excluded from the final analysis. A total of 1871 individuals received the online questionnaire. Of those, 1800 participants provided informed consent and completed the survey, constituting the total completed-survey sample, which included 884 Muslim and 916 Jewish participants aged 18–81 years, representing diverse levels of religiosity, education, socio-economic status, and geographic distribution. The Muslim and Jewish subsamples were previously examined separately [62,63]. The present study uses the same underlying dataset and necessarily repeats selected descriptive information, but its direct interfaith comparisons, tests of differences between coefficients, and multi-group path and mediation analyses are new and novel contributions.

### 2.2. Recruitment and Sampling

Recruitment was conducted through a multiplatform social media strategy aimed at reaching geographically and demographically diverse Muslim and Jewish populations in Israel. The online questionnaire was distributed via Facebook, WhatsApp, and Instagram using convenience and snowball sampling through community groups, religious and cultural forums, academic and professional networks, and the research team’s personal contacts. To enhance accessibility, recruitment materials were provided in Hebrew and Arabic, and participants were encouraged to share the survey within their networks. Furthermore, geographic representation was ensured by including a mandatory questionnaire item in which participants reported their region of residence (North, Center, or South of Israel). This enabled verification of the sample’s regional distribution and ensured that respondents from all predefined regions were adequately represented.

### 2.3. Questionnaire Development and Validation

The questionnaire was developed through a structured process informed by established methodological recommendations for questionnaire design and preliminary evaluation [64,65]. The study was conducted in two phases: (1) an exploratory pilot study and (2) the main cross-sectional study. Two distinct constructs were operationalized: “Knowledge,” referring to participants’ perceptions of whether their religion permits the specified medical uses, and “Opinion,” referring to participants’ personal views regarding whether their religion should permit such uses. Content validity was assessed by an interdisciplinary panel of five experts in medical ethics, transplantation, and health research, who evaluated the relevance and clarity of the items and their alignment with the study objectives. During the pilot phase, face validity was assessed to confirm item comprehensibility and cultural appropriateness. Pilot data were used to examine internal consistency and explore the questionnaire’s item structure, leading to minor refinements before administration in the main study. The pilot sample of 50 participants yielded a Cronbach’s alpha of 0.76. The questionnaire was developed in Arabic and subsequently translated into Hebrew. Bilingual experts reviewed both versions for semantic equivalence, linguistic accuracy, cultural appropriateness, and preservation of the intended conceptual meaning. In the main study sample, Cronbach’s α for the six-item Knowledge scale was 0.988 among Muslim participants (*n* = 728) and 0.982 among Jewish participants (*n* = 806). For the six-item Opinion scale, α was 0.979 among Muslims (*n* = 884) and 0.981 among Jews (*n* = 916). Coefficients were calculated separately by group using complete item sets within each scale. “I don’t know” responses were treated as missing in the Knowledge reliability analysis; therefore, its sample sizes differ from those used for the available-item Knowledge composite.

### 2.4. Data Collection and Study Tool

Using the pilot findings, we administered an online questionnaire in Arabic and Hebrew from 1 February 2023 to 31 December 2024 (Supplementary File S1). The instrument contained three sections: (1) demographic characteristics, including age, gender, education, religious affiliation, religiosity, marital status, and number of children; (2) perceived knowledge of religious permissibility; and (3) opinions regarding the permissibility of six porcine-derived procedures involving the heart or heart valve, lung, kidney, pancreas or pancreatic cells, knee cartilage, and skin. For each procedure, the knowledge item included six ordered permissibility categories (1–6) and a separate “I don’t know” option coded 99; the corresponding opinion item used a 7-point agreement scale. The study did not classify participants according to whether their opinions were genuinely positive; it measured only the attitudes they reported in response to the anonymous questionnaire.

### 2.5. Ethical Approval

The study was reviewed and approved by the Ethics Committee of the Academic College of Tel Aviv-Yaffo, Israel (Protocol No. 2022-1081; 31 December 2022), and all procedures followed the Declaration of Helsinki. Before accessing the questionnaire, participants viewed an electronic informed-consent form describing the study purpose, the nature of the questions, the approximate 15-min completion time, voluntary participation, anonymity and confidentiality safeguards, the absence of expected direct personal benefit, the minimal privacy risk associated with online participation, and the right to discontinue at any time without consequence. Research-team contact details were provided. Only individuals aged 18 years or older who selected “I agree” could proceed. Because this was an anonymous, non-interventional online survey with no treatment allocation or assessor-rated outcome, blinding was not applicable.

### 2.6. Data Analysis

For a two-sided independent-samples T test with equal allocation, Cohen’s d = 0.20, α = 0.05, and 80% power, the required sample size using G*Power 3.1 (Heinrich Heine University Düsseldorf, Düsseldorf, Germany) is approximately 394 participants per group. A larger recruitment target of more than 800 participants per group was set before data collection to increase statistical power beyond this minimum requirement. The final sample comprised 884 Muslim and 916 Jewish participants. This calculation applies to between-group mean comparisons and does not establish power for every regression coefficient or indirect effect.

Knowledge responses were scored using the six ordered categories coded 1–6. The separate “I don’t know” response, coded 99, was treated as missing rather than as a numerical scale value. Item means and standard deviations excluded these responses on an item-specific basis. The composite Knowledge index was the mean of the available valid responses across the six items, requiring at least one valid response; no missing values were imputed. Organ-specific Knowledge–Opinion correlations used valid response pairs for the corresponding items. Path and indirect-effect analyses included participants with a calculable Knowledge composite and complete data on the remaining model variables, using identical cases in both regression equations within each group. Participants with all six Knowledge responses coded 99 had no composite score and were excluded from these analyses: 112 Muslims and 70 Jews, leaving analytic samples of 772 and 846, respectively.

All analyses were conducted on composite scale scores (mean indices for knowledge and opinion across the six scenarios), which are commonly treated as approximately continuous in larger samples. Normality of these composite variables was assessed using the Kolmogorov–Smirnov test and visual inspection (histograms and Q–Q plots), which indicated a normal distribution for the continuous variables in our study, with no meaningful deviations; thus, parametric analyses were used, including *t*-tests, Pearson correlations, and MANOVA. Continuous scores were summarized using means and standard deviations. Between-group mean comparisons used two-sided Welch independent-samples t tests and Cohen’s d, defined as Jewish minus Muslim divided by the pooled within-group standard deviation. The two composite mean comparisons were reported separately. Pearson correlations used valid Knowledge–Opinion response pairs for each organ; Fisher’s Z compared the corresponding correlations between independent groups. All between-group t and Z contrasts were calculated as Jewish minus Muslim. A nominal *p* < 0.05 was used for exploratory comparisons; nominal findings were distinguished from multiplicity-adjusted results.

Opinion was the mean of all six valid 1–7 responses. Cronbach’s α was computed separately by group and scale using complete item sets; therefore, Knowledge reliability used a stricter sample than the available-item composite.

Multi-group path model specified six demographic predictors of Knowledge and six direct demographic paths to Opinion, together with Knowledge → Opinion. Both equations used the same 772 Muslim and 846 Jewish participants. Within each group, 21 predictor variance/covariance parameters, 13 directed coefficients, and two residual variances matched the 36 observed covariance moments (df = 0). Predictor means and regression intercepts were group-specific, and the residual covariance between the two equations was fixed to zero. A nested conditional Gaussian maximum-likelihood comparison constrained all 13 corresponding unstandardized coefficients to equality across groups, retaining the other parameters as group-specific (Δdf = 13). A supplementary HC3 joint Wald test assessed all-path equality under heteroskedasticity. This evaluates structural-path equality, not measurement invariance of latent constructs.

Bootstrapping was applied in mediation analyses to ensure robustness to potential non-normality. Path analyses with multiple-group comparisons were conducted to examine mediation models, with demographic variables as predictors, knowledge as the mediator, and opinion as the outcome. The bootstrapping method (5000 resamples, 95% bias-corrected confidence intervals) was employed to evaluate mediation effects. Between-group differences in individual path coefficients were assessed using Z tests. Statistical significance was set at *p* < 0.05. Analyses were conducted using SPSS version 28 and AMOS version 31 (IBM, Armonk, NY, USA).

## 3. Results

Overall, 1800 participants (49.1% men) aged 18–81 years (mean = 50.7, SD = 20.6) were enrolled: 884 Muslim (49.1%) and 916 Jewish (50.9%) participants; additionally, the two groups were similar in gender distribution (Muslims: 48.3% male, 51.7% female; Jews: 49.9% male, 50.1% female). The mean number of children was 4.4 (SD = 2.8) vs. 4.0 (SD = 2.7) in Muslim vs. Jewish participants; the respective percentage of secular individuals was 45.5% vs. 49.1%, and that of individuals having an academic education was 50.3% vs. 52.5%. Furthermore, most of the participants were in a relationship (e.g., married): 90.3% vs. 82.9% in Muslim vs. Jewish participants, respectively (Table 1).

Across all six items assessing knowledge and opinion toward porcine-derived organ transplantation, both Jewish and Muslim participants exhibited moderate levels of acceptance, with substantial variability depending on the specific organ. Table 2 presents organ-specific Knowledge and Opinion scores and their between-group comparisons. Welch independent-samples *t* tests indicated that Muslim participants had higher mean Knowledge scores concerning skin grafting than Jewish participants (M = 2.05 vs. 1.85; t (1445.5) = −2.52, *p* = 0.012; Cohen’s d = −0.130). The remaining organ-specific Knowledge mean differences were not statistically significant.

For Opinion, Muslim participants reported higher mean scores concerning lung transplantation (M = 3.45 vs. 3.11; t (1722.0) = −3.08, *p* = 0.002; d = −0.146), knee cartilage replacement (M = 2.47 versus 2.21; t (1660.9) = −2.83, *p* = 0.005; d = −0.134), and skin grafting (M = 2.13 versus 1.95; t (1657.8) = −2.27, *p* = 0.023; d = −0.108). Opinion mean differences for heart/heart-valve, kidney, and pancreas/pancreatic-cell transplantation were not statistically significant. All observed standardized mean differences were small in magnitude (Table 2).

Knowledge and Opinion were positively correlated for all six procedures in both groups. Fisher’s Z tests compared these correlations between groups, rather than their mean scores. Correlations were stronger among Muslims for heart/heart-valve and kidney transplantation, and stronger among Jews for pancreas/pancreatic-cell transplantation, knee cartilage replacement, and skin grafting. The between-group correlation difference for lung transplantation was not statistically significant (Z = −0.83, *p* = 0.405) (Table 2).

Table 3 presents differences in the demographic associations with Knowledge and Opinion between Muslim and Jewish participants. Age was negatively associated with Knowledge among Muslims (β = −0.73) and positively associated among Jews (β = 0.23), with a significant between-group difference in the corresponding unstandardized coefficients (Z = 22.26, *p* < 0.001). Number of children also showed opposite directions of association (Muslims: β = −0.27; Jews: β = 0.585; Z = 16.29, *p* < 0.001). Religious identification was positively associated with Knowledge in both groups, with standardized coefficients of 0.05 among Muslims and 0.52 among Jews (Z = 15.02, *p* < 0.001). Female gender was negatively associated with Knowledge in both groups (Muslims: β = −0.03; Jews: β = −0.10), and the between-group coefficient comparison yielded Z = −2.72, *p* = 0.007. In contrast, marital status showed small negative coefficients in both groups (β = −0.003 and −0.06, respectively), and their difference was not statistically significant (Z = −1.64, *p* = 0.101).

In the separate demographic regressions predicting Opinion without Knowledge as a predictor, age was negatively associated with Opinion among Muslims (β = −0.61) and positively associated among Jews (β = 0.43; Z = 25.96, *p* < 0.001). Education showed a small negative coefficient among Muslims and a positive coefficient among Jews (β = −0.02 and 0.23, respectively; Z = 11.36, *p* < 0.001). Religious identification was positively associated with Opinion in both groups (Muslims: β = 0.03; Jews: β = 0.53; Z = 19.65, *p* < 0.001). Being in a relationship showed negative coefficients in both groups (β = −0.12 and −0.02, respectively; Z = 6.07, *p* < 0.001). Number of children was negatively associated with Opinion among Muslims and positively associated among Jews (β = −0.31 and 0.37, respectively; Z = 15.07, *p* < 0.001).

In the additional model adjusting for all six demographic predictors, Knowledge was strongly and positively associated with Opinion among both Muslims (β = 0.95) and Jews (β = 0.79). The between-group comparison of the corresponding unstandardized coefficients yielded Z = −2.04 and *p* = 0.041. These findings concern perceived religious permissibility and stated opinions, rather than objectively assessed factual knowledge or actual treatment decisions.

Furthermore, path analysis with multiple-group comparisons was used to examine a mediation model in which demographic characteristics were predictors, Knowledge was the mediator, and Opinion was the outcome. To examine indirect associations between demographic characteristics and Opinion through Knowledge, an observed-variable multi-group path model was estimated for 772 Muslim and 846 Jewish participants with calculable Knowledge composite scores and complete data on the remaining model variables. The model included paths from all six demographic predictors to both Knowledge and Opinion, together with a Knowledge → Opinion path. The unconstrained model freely estimated all 13 structural coefficients within each group and was saturated (df = 0); therefore, global fit indices were not interpreted. A nested model constrained the 13 corresponding unstandardized coefficients to equality across groups while retaining group-specific predictor means and variances/covariances, regression intercepts, and residual variances. The likelihood-ratio comparison rejected equality of the complete set of structural coefficients (Δχ^2^ (13) = 2718.19, *p* < 0.001), indicating that at least some structural associations differed between groups. This omnibus result does not establish that every individual path or indirect association differed between groups.

Table 4 presents the mediation analyses examining the fully standardized indirect associations between demographic predictors and Opinion through Knowledge, based on 772 Muslim and 846 Jewish participants. Among Muslims, negative indirect associations were observed for age (Eff. = −0.687, 95% bias-corrected CI [−0.741, −0.635]), female gender (Eff. = −0.031, CI [−0.053, −0.009]), academic education (Eff. = −0.043, CI [−0.069, −0.018]), and number of children (Eff. = −0.259, CI [−0.314, −0.201]). Religious identification showed a positive indirect association (Eff. = 0.052, CI [0.016, 0.087]). The indirect association for being in a relationship was not statistically significant, as its confidence interval included zero (Eff. = −0.003, CI [−0.015, 0.008]; *p* = 0.619).

Among Jews, the confidence intervals excluded zero for all six indirect associations. Positive indirect associations were observed for age (Eff. = 0.185, CI [0.129, 0.246]), academic education (Eff. = 0.261, CI [0.223, 0.298]), religious identification (Eff. = 0.412, CI [0.373, 0.447]), and number of children (Eff. = 0.455, CI [0.387, 0.515]). Negative indirect associations were observed for female gender (Eff. = −0.080, CI [−0.110, −0.049]) and being in a relationship (Eff. = −0.050, CI [−0.088, −0.012]).

These results provide evidence of indirect associations through Knowledge for five demographic predictors among Muslims and all six predictors among Jews. Age, education, and number of children showed opposite directions of indirect association across the groups. These descriptive differences do not, by themselves, establish statistically significant between-group differences in the indirect effects. Furthermore, the cross-sectional design does not establish temporal ordering or causal mediation; the findings should therefore be interpreted as indirect statistical associations rather than evidence that Knowledge causally mediates demographic influences on Opinion.

## 4. Discussion

Islam and Judaism prohibit pork and view pigs as ritually impure, shaping attitudes toward porcine-derived medical treatments [2,41,54]. While religious faith may aid coping, it can also lead to refusal of such treatments unless justified through principles such as Istihala [2,25,41,46].

The religious considerations identified in Muslim and Jewish contexts align with findings from other cultural settings, underscoring the broader impact of faith, education, and socio-cultural frameworks on attitudes toward xenotransplantation. Studies from Turkey [4,5,6,7] demonstrate that religious awareness and cultural norms significantly shape public and patient acceptance of XTx, even among individuals already engaged in transplant care. However, Dogan et al. [6] highlighted the impact of educational background, which could help explain intra-group differences in our study. These findings are consistent with our observations and emphasize that addressing religious and ethical concerns is as essential as overcoming technical challenges.

The novelty of our study lies in its combination of a direct interfaith comparison, a broad examination of multiple organ types, and the integration of theological, cultural, and ethical perspectives with quantitative statistical modeling, including path analysis. This approach offers a new framework for understanding how religious knowledge shapes healthcare decisions, with direct implications for culturally sensitive informed consent in transplantation medicine. Our findings reveal limited public awareness in both groups regarding the broad religious permissibility of life-saving organ transplantation using pig-derived organs. Jewish participants demonstrated higher levels of knowledge and stronger beliefs in the permissibility of porcine-derived organ use, and they also perceived greater communal awareness of this permissibility compared with Muslim participants.

In both groups, mean Knowledge and Opinion scores were highest for heart/heart-valve transplantation, followed by kidney, lung, pancreas/pancreatic cells, knee cartilage, and skin transplantation. These rankings describe relative differences across the hypothetical clinical scenarios. They do not indicate high overall support: all organ-specific Opinion means were below the midpoint of the seven-point scale in both groups.

Previous studies carried out among the Jewish population regarding the use of porcine products indicated higher rankings of heart valve and knee cartilage transplantation and post-burn skin graft in order of priority than among the Muslim population [22,27,60].

The Muslim respondents had a lower level of belief as to the permissibility of all the transplants presented in the questionnaire. These findings highlight the importance for physicians, surgeons, and transplant coordinators to be attentive to patient-specific concerns, as issues that may be perceived by healthcare professionals as marginal or clinically irrelevant can nevertheless carry substantial meaning for patients [20]. For some patients, fulfilling their religious, cultural, and ethical beliefs may be more important than achieving an improvement in their physical health [53]. In some cases, the patient may wish to consult with a clergyman or religious leader. The medical staff must respect this request and delay treatment insofar as possible until the patient feels that they have received the appropriate advice [23,66].

The positive Pearson correlations and path-analysis coefficients between the Knowledge and Opinion indices indicate that participants who perceived a porcine-derived intervention as religiously permissible were more likely to support its use. Across the six organ-specific pairs, the within-group correlations ranged from 0.79 to 0.96, indicating consistently strong associations, although their magnitude varied across organs and study groups. These associations should be interpreted cautiously because knowledge and opinion were measured within the same questionnaire, in immediate succession, using parallel organ-specific items; shared item wording and common-method variance may therefore have inflated the observed relationships.

Previous studies suggest that familiarity with organ donation, personal or indirect experience, knowledge, and the immediate clinical context may influence attitudes and willingness regarding organ donation and transplantation [67,68,69]. Accordingly, attitudes may differ when an individual or a close family member faces an actual need for transplantation. However, because participants’ personal or familial experience with, or need for, transplantation was not assessed, this possibility could not be examined in the present study. Future studies should investigate whether attitudes differ according to personal or familial need for transplantation and prior exposure to transplant-related care.

### 4.1. Interpretation of Differences in Knowledge and Attitudes

The differences observed between Jewish and Muslim participants in both knowledge and attitudes toward porcine-derived medical interventions likely reflect the influence of multiple religious, cultural, and informational determinants rather than a single explanatory factor. In the Jewish context, the halachic principle of “*pikuach nefesh*” the obligation to preserve human life, has long been a central, widely disseminated norm within both religious and clinical discourse. This broader familiarity may help explain the higher levels of reported knowledge and the more favorable attitudes observed in this group [53,54].

In Islamic jurisprudence, the doctrine of *darūrah* (necessity) similarly allows the use of otherwise prohibited substances when required to preserve life. This reliance on individualized interpretation may contribute to greater uncertainty among Muslim participants, particularly when clear guidance is not readily accessible through trusted religious or medical channels [2,25,26,41]. The weaker association between knowledge and attitudes among Muslim respondents observed in the SEM analysis indicates that biomedical information alone exerts limited influence, with attitudes shaped more strongly by religious interpretation and communal norms. Low knowledge levels across groups point to systemic rather than individual gaps. These findings underscore structural and communicative limitations at the intersection of medicine, religion, and public health, suggesting the need for coordinated efforts by healthcare providers, religious authorities, and patient-centered educational initiatives.

This study has several limitations. Online convenience and snowball sampling may have produced selection bias by over-representing digitally active networks, limiting representativeness. Although anonymous data collection likely reduced social desirability bias, it could not eliminate it. Therefore, the findings reflect self-reported attitudes, which may not fully represent privately held views or actual behavior.

Knowledge–opinion correlations should be interpreted cautiously because both constructs were measured within the same questionnaire using parallel, organ-specific items presented in immediate succession. Moreover, the measures were self-reported and reflected participants’ responses to hypothetical scenarios rather than actual decisions made when transplantation was clinically required. Social desirability, similarities in item wording, and the sequential presentation of the knowledge and opinion items may therefore have introduced common-method variance and inflated the observed correlations.

Religious beliefs and their interpretation are heterogeneous within both Judaism and Islam. The present comparison should not be interpreted as establishing that one tradition is uniformly more restrictive or permissive than the other. The findings reflect this Israeli sample and may not generalize to Muslim and Jewish communities in other countries or to individuals who follow different denominational, cultural, or scholarly interpretations.

The cross-sectional design precludes causal inference. The observed associations between perceived religious knowledge and attitudes should be interpreted as correlational.

This study did not assess whether participants or their close associates had previously needed, received, or donated an organ, or whether they were currently facing a need for transplantation. Consequently, the possible influence of actual medical necessity on transplantation attitudes could not be evaluated. Prior exposure and immediate medical need could materially alter acceptance and should be examined in future prospective, qualitative, and mixed-methods studies involving transplant candidates and their families. Quantitative survey items may not capture all ethical, emotional, and religious nuances relevant to an actual treatment decision.

The strengths of our study include a large sample with balanced representation across population groups, a wide age range, and inclusion of both sexes, with integration of multiple research methodologies and validation of the questionnaire through a pilot study using face and content validity, which provided the basis for constructing the final version used in our study.

### 4.2. Implications for Clinical Practice

The SEM analyses revealed meaningful differences between Muslim and Jewish participants in how demographic factors and religious knowledge relate to attitudes toward porcine-derived xenotransplantation. These differences carry implications for clinical practice, particularly regarding patient education and informed consent.

Among Jewish participants, knowledge was more strongly associated with favorable attitudes toward porcine-derived organ use, indicating that clarifying relevant rabbinical guidance may effectively increase acceptance. Among Muslim participants, this association was weaker, with demographic and religiosity-related factors exerting greater influence. Thus, discussions with Muslim patients may require not only factual explanation but also engagement with religious interpretation and opportunities for consultation with religious authorities.

These findings emphasize the need for culturally adapted communication. Clinicians should avoid uniform messaging and tailor discussions to patients’ religious and cultural contexts. For some, clarifying medical necessity and religious permissibility may suffice; for others, consultation with spiritual authorities may be essential. Clinicians should incorporate a brief, structured assessment before recommending or administering an animal-derived treatment. Patients should first be asked directly whether the animal origin of medicines, implants, or biological materials could influence their treatment decision. If the proposed intervention contains porcine-derived material, the clinician should identify the specific product and its source, explain why it is medically indicated, clarify whether the situation is urgent or whether a decision can be deferred, and discuss the expected benefits, relevant risks, and any clinically appropriate non-porcine alternatives. Patient understanding should then be confirmed using a teach-back question, and the patient’s stated concerns, preferences, and final decision should be documented in the medical record. When treatment can be safely delayed, patients requesting religious guidance should be offered an opportunity to consult a trusted religious authority and, if desired, a family member. Such alignment with the pathways identified in the SEM analyses supports informed decision-making and promotes patient-centered, ethically grounded care in recommending porcine-derived treatments.

This study advances the field by integrating theological perspectives from Judaism and Islam with empirical analysis of how religious knowledge shapes attitudes toward organ donation. By combining ethical, cultural, and statistical insights, it helps bridge the gap between religious principles and their practical implications for patient-centered transplant care. Both traditions allow exceptions in life-threatening situations, underscoring the importance of dialogue between religious authorities and transplant teams. Ensuring culturally sensitive care requires religious leaders to remain informed about relevant medical technologies, while clinicians must respect patients’ beliefs and facilitate consultation with religious authorities when needed.

Recent developments in XTx provide grounds for cautious optimism. In March 2024, physician-scientists at Harvard Medical School and Massachusetts General Hospital successfully transplanted a genetically modified pig kidney into a patient with type 2 diabetes and hypertension. Although the patient survived for only two months, his death was not linked to the transplantation itself. This achievement nonetheless represents a significant milestone in efforts to address the severe shortage of human kidneys available for patients with end-stage renal disease, offering hope for future advances in the field [70]. Healthcare providers should demonstrate sensitivity to the religious beliefs of all patients and ensure that they receive comprehensive, relevant, and accessible information regarding their care [63,71]. By fostering transparency, clinicians enable patients to make truly informed decisions about their treatment in ways that respect and integrate their physical, emotional, spiritual, cultural, and religious values.

## 5. Conclusions

This study identifies limited awareness of religious considerations surrounding life-saving porcine-derived transplantation among Muslim and Jewish participants. Greater perceived religious knowledge was associated with more favorable attitudes, although the strength of this relationship differed between groups and may partly reflect common-method effects. These findings support culturally sensitive education and informed-consent processes that explain medical necessity and relevant religious perspectives without presuming an individual patient’s beliefs. Dialogue with a qualified religious authority should be facilitated when requested, while the patient’s autonomy remains central. Strong Knowledge–Opinion associations require caution about common-method effects and do not establish causal mediation or actual treatment choices.

## Figures and Tables

**Table 1:** Demographic data for Muslim and Jewish participants.

Variable	Category	Nm (Nj)	%m (%j)	Mm (Mj)	SDm (SDj)	Rm (Rj)
Gender	Male	427 (457)	48.3 (49.9)	-	-	-
	Female	457 (459)	51.7 (50.1)	-	-	-
Education	Not academic	439 (435)	49.7 (47.5)	-	-	-
	Academic	445 (481)	50.3 (52.5)	-	-	-
Religiosity	Secular	402 (450)	45.5 (49.1)	-	-	-
	Religious	482 (466)	54.5 (50.9)	-	-	-
Marital Status	No relationship	86 (157)	9.7 (17.1)	-	-	-
	In a relationship	798 (759)	90.3 (82.9)	-	-	-
Age		-	-	49.41 (51.13)	20.43 (20.78)	18–81
Number of Children		-	-	4.43 (3.96)	2.84 (2.73)	0–13 (0–10)

The letters m and j indicate the different samples, where m = Muslim sample and j = Jewish sample. N = frequency. % = relative percentage. M = mean. SD = standard deviation. R = range. For regression analyses, the questionnaire categories were recoded consistently as follows: gender, 0 = male and 1 = female; education, 0 = not academic and 1 = academic; religiosity, 0 = secular and 1 = religious; marital status, 0 = no relationship and 1 = in a relationship.

**Table 2:** Means, standard deviations, Pearson zero-order correlations, and difference tests for all Knowledge and Opinion items.

	Jewish (Total *n* = 916)			Muslim (Total *n* = 884)			r Test	Between-Group Mean Comparisons ^1^	
Item	M_k_ (M_o_)	SD_k_ (SD_o_)	r_p_	M_k_ (M_o_)	SD_k_ (SD_o_)	r_p_	Fisher Z *p*	Welch t (df), *p* K/O	Cohen d K/O
1. Transplanting a heart or a heart valve taken from a pig into a patient suffering from a heart problem whose life is in danger	3.18 (3.79)	1.86 (2.28)	0.932	3.14 (3.81)	1.95 (2.51)	0.966	−7.07 <0.001	0.46 (1586.4), *p* = 0.648 −0.12 (1767.8), *p* = 0.901	0.023 −0.006
2. Transplanting a lung taken from a pig in a patient suffering from severe obstructive pulmonary disease	2.64 (3.11)	1.80 (2.09)	0.958	2.80 (3.45)	1.88 (2.49)	0.961	−0.83 0.405	−1.73 (1571.2), *p* = 0.083 −3.08 (1722.0), *p* = 0.002	−0.087 −0.146
3. Transplanting a kidney taken from a pig in a patient suffering from severe renal insufficiency	2.99 (3.46)	1.86 (2.17)	0.934	2.91 (3.55)	1.94 (2.53)	0.957	−4.38 <0.001	0.83 (1553.7), *p* = 0.408 −0.85 (1736.5), *p* = 0.396	0.042 −0.040
4. Transplanting a pancreas/pancreatic cells taken from a pig in a patient who is in danger of death from pancreatic insufficiency	2.38 (2.73)	1.77 (1.94)	0.954	2.40 (2.82)	1.82 (2.35)	0.941	2.45 0.014	−0.13 (1542.9), *p* = 0.896 −0.94 (1713.9), *p* = 0.349	−0.007 −0.044
5. Transplanting a patient’s knee cartilage taken from a pig to replace worn cartilage	2.07 (2.21)	1.58 (1.68)	0.965	2.22 (2.47)	1.74 (2.18)	0.879	12.49 <0.001	−1.79 (1494.5), *p* = 0.074 −2.83 (1660.9), *p* = 0.005	−0.091 −0.134
6. Using skin tissue of a pig to do a skin graft in a patient with serious burn injuries	1.85 (1.95)	1.42 (1.50)	0.958	2.05 (2.13)	1.65 (1.95)	0.788	16.58 <0.001	−2.52 (1445.5), *p* = 0.012 −2.27 (1657.8), *p* = 0.023	−0.130 −0.108

M = mean; SD = standard deviation; k/K = Knowledge; o/O = Opinion; r_p_ = Pearson correlation between the corresponding Knowledge and Opinion items within each group. Parentheses in the M and SD columns contain Opinion values. In the two added mean-comparison columns, the first line gives Knowledge values, and the second gives Opinion values. ^1^ All between-group contrasts are Jewish minus Muslim. Welch t tests compare means, using unequal-variance standard errors and fractional degrees of freedom. Cohen d uses the pooled within-group SD. Negative t or d indicates a higher Muslim mean. The displayed *p* values are two-sided and nominal (unadjusted for multiple comparisons). Fisher Z compares Fisher-transformed Knowledge–Opinion correlations and Jewish minus Muslim; its *p* values are nominal and do not test means. Knowledge means and correlations use item-specific valid samples. Valid n for items 1–6: Jewish 846, 838, 830, 822, 814, 806; Muslim 772, 764, 755, 747, 738, 728. Opinion means and tests use all 916 Jewish and 884 Muslim participants. Knowledge has six ordered categories coded 1–6 and a separate “I don’t know” response coded 99. Code 99 is excluded from Knowledge means and correlations. *Likert scale options for knowledge*: (1) It is 100% forbidden, (2) It is almost always forbidden, (3) It is permitted infrequently, (4) It is permitted in exceptional cases, (5) It is almost always permitted, (6) It is totally 100% permitted, (99) I don’t know. *Likert scale options for opinion*: (1) Totally disagree, (2) Disagree somewhat, (3) Agree slightly, (4) Agree moderately, (5) Agree to a large extent, (6) Agree strongly, (7) Totally agree.

**Table 3:** Path analysis results with standardized regression coefficients and difference tests.

			Muslims (*n* = 884)	Jews (*n* = 916)	Difference Test	
Path			β	β	*Z*-Scores	*p* Value
Age	→	Knowledge ^1^	−0.73	0.23	22.26	<0.001
Gender	→	Knowledge ^1^	−0.03	−0.10	−2.72	0.007
Education	→	Knowledge ^1^	−0.05	0.33	13.96	<0.001
Religiosity	→	Knowledge ^1^	0.05	0.52	15.02	<0.001
Marital Status	→	Knowledge ^1^	−0.003	−0.06	−1.64	0.101
Children	→	Knowledge ^1^	−0.27	0.58	16.29	<0.001
Age	→	Opinion ^2^	−0.61	0.43	25.96	<0.001
Gender	→	Opinion ^2^	−0.03	−0.08	−2.03	0.042
Education	→	Opinion ^2^	−0.02	0.23	11.36	<0.001
Religiosity	→	Opinion ^2^	0.03	0.53	19.65	<0.001
Marital Status	→	Opinion ^2^	−0.12	−0.02	6.07	<0.001
Number of Children	→	Opinion ^2^	−0.31	0.37	15.07	<0.001
Knowledge	→	Opinion ^3^	0.95	0.79	−2.04	0.041

β = standardized regression coefficient; *n* = sample size. Model ^1^ (demographic predictors → Knowledge): all six demographic predictors were entered simultaneously; *n* = 772 Muslims and 846 Jews. Model ^2^ (demographic predictors → Opinion): all six demographic predictors were entered simultaneously without Knowledge; all 884 Muslims and 916 Jews were included. Model ^3^ (Knowledge → Opinion, final row): Knowledge was entered together with all six demographic predictors; *n* = 772 Muslims and 846 Jews. Thus, demographic → Opinion rows and the final Knowledge → Opinion row come from different models. Z compares corresponding unstandardized coefficients between independent groups: Z = (B Jewish − B Muslim)/√(SE Jewish^2^ + SE Muslim^2^). *p* values are two-sided standard-normal probabilities and are nominal, without adjustment for multiple comparisons; the displayed standardized β values are descriptive. Coding: gender, 0 = male, 1 = female; education, 0 = non-academic, 1 = academic; religiosity, 0 = secular, 1 = religious; marital status, 0 = no relationship, 1 = in a relationship. Original religiosity codes 1 = secular and 2 = religious were recoded as 0 and 1. Age and number of children are continuous.

**Table 4:** Mediation (indirect) effects analyses.

					Muslims (*n* = 772)				Jews (*n* = 846)			
Path					Eff.	LL	UL	*p* Value	Eff.	LL	UL	*p* Value
Age	→	Knowledge	→	Opinion	−0.687	−0.741	−0.635	<0.001	0.185	0.129	0.246	<0.001
Gender	→	Knowledge	→	Opinion	−0.031	−0.053	−0.009	0.007	−0.080	−0.110	−0.049	<0.001
Education	→	Knowledge	→	Opinion	−0.043	−0.069	−0.018	<0.001	0.261	0.223	0.298	<0.001
Religiosity	→	Knowledge	→	Opinion	0.052	0.016	0.087	0.004	0.412	0.373	0.447	<0.001
Marital Status	→	Knowledge	→	Opinion	−0.003	−0.015	0.008	0.619	−0.050	−0.088	−0.012	0.010
Number of children	→	Knowledge	→	Opinion	−0.259	−0.314	−0.201	<0.001	0.455	0.387	0.515	<0.001

Analyses used bootstrapping (95% bias-corrected confidence intervals; 5000 resamples).  *p* values are nominal, determined using two-sided normal-approximation Wald tests of the unstandardized indirect effect. *p* values are not adjusted for multiple testing. Eff. = standardized indirect effect (predictor → Knowledge → Opinion); LL = lower limit; UL = upper limit; Children = number of children. Gender: 0 = male, 1 = female; education: 0 = non-academic, 1 = academic; religiosity: 0 = secular, 1 = religious; marital status: 0 = no relationship, 1 = in a relationship. Age and number of children are continuous. Indirect associations in this cross-sectional study do not establish causal mediation.

## Data Availability

The data presented in this study are available on request from the corresponding author due to privacy restrictions.

## Supplementary Materials

The following supporting information can be downloaded at https://www.mdpi.com/article/10.3390/healthcare14213614/s1, File S1: The research questionnaire.

## Abbreviations

The following abbreviations are used in this manuscript:

β	Standardized regression coefficient
B	Unstandardized regression coefficient
BC	Bias-corrected bootstrap interval
HC3	Heteroskedasticity-consistent covariance estimate
CI	Confidence interval
Eff.	Standardized indirect effect
Mk	Mean of knowledge
Mo	Mean of opinion
rp	Pearson correlation coefficient
SD	Standard deviation
SEM	Structural equation modeling
XTx	Xenotransplantation
