# Supplementary material for: Xenotransplantation of Porcine-Derived Products: Attitudes of Muslims and Jews Based on Religion, Culture, Ethical Views, and Perceived Knowledge
Source: Healthcare (Basel). 2026 Oct 26;14(21):3614. doi: 10.3390/healthcare14213614 (PMC13763084; doi:10.3390/healthcare14213614)
Supplement: Supplementary file 1 [file healthcare-14-03614-s001.zip › healthcare-4557769-supplementary.pdf]

File S1: The research questionnaire

1 -Age (in years):

2 -Gender: 1 - Male 2- Female

3 -Education: 1. Academic 2. Non-academic

4 -Religion: 1. Muslim 2. Jew

5 -Level of religiosity: 1- Religious, 2- Secular

6 -Family status: 1. In a relationship 2. Not in a relationship

7. Number of children…….

8. Region of residence: 1. North 2. Center 3. South

The first part of the questionnaire presents you with several medical treatment scenarios. Please indicate, to the best of your knowledge, to what extent the Jewish/Islamic religion allows medical treatment as described in the scenario:

1<br>It is 100% forbidden	2<br>It is almost always forbidden	3<br>It is permitted infrequently	4<br>It is permitted in exceptional cases	5<br>It is almost always permitted	6<br>It is totally 100% permitted	99<br>I don't know

1. Is it permitted according to the Jewish/Islamic religion to transplant a heart, or a heart valve taken from a pig into a patient suffering from a heart problem whose life is in danger?

1<br>It is 100% forbidden	2<br>It is almost always forbidden	3<br>It is permitted infrequently	4<br>It is permitted in exceptional cases	5<br>It is almost always permitted	6<br>It is totally 100% permitted	99<br>I don't know

2. Is it permitted according to the Jewish/Islamic religion to do a lung transplant in a patient suffering from severe obstructive pulmonary disease, using a lung taken from a pig?

1<br>It is 100% forbidden	2<br>It is almost always forbidden	3<br>It is permitted infrequently	4<br>It is permitted in exceptional cases	5<br>It is almost always permitted	6<br>It is totally 100% permitted	99<br>I don't know

3. Is it permitted according to the Jewish/Islamic religion to do a kidney transplant in a patient suffering from severe renal insufficiency using a kidney taken from a pig?

1<br>It is 100% forbidden	2<br>It is almost always forbidden	3<br>It is permitted infrequently	4<br>It is permitted in exceptional cases	5<br>It is almost always permitted	6<br>It is totally 100% permitted	99<br>I don't know

4. Is it permitted according to the Jewish/Islamic religion to treat a patient who is in danger of death from pancreatic insufficiency by doing a transplant of a pancreas/pancreatic cells taken from a pig?

1<br>It is 100% forbidden	2<br>It is almost always forbidden	3<br>It is permitted infrequently	4<br>It is permitted in exceptional cases	5<br>It is almost always permitted	6<br>It is totally 100% permitted	99<br>I don't know

5. Is it permitted according to the Jewish/Islamic religion to implant in a patient's knee cartilage taken from a pig, to replace worn cartilage?

1<br>It is 100% forbidden	2<br>It is almost always forbidden	3<br>It is permitted infrequently	4<br>It is permitted in exceptional cases	5<br>It is almost always permitted	6<br>It is totally 100% permitted	99<br>I don't know

6. Is it permitted according to the Jewish/Islamic religion to use the skin tissue of a pig to do a skin graft in a patient with serious burn injuries?

1<br>It is 100% forbidden	2<br>It is almost always forbidden	3<br>It is permitted infrequently	4<br>It is permitted in exceptional cases	5<br>It is almost always permitted	6<br>It is totally 100% permitted	99<br>I don't know

In the second part of the questionnaire, you are asked to read each scenario and state your personal opinion on whether and to what extent you support providing the medical treatment described in the scenario.

7. Do you think one should approve transplanting in a Jewish/Muslim patient a heart or a heart valve from a pig into a Jewish/Muslim patient?

1<br>Totally disagree	2<br>Disagree somewhat	3<br>Agree slightly	4<br>Agree moderately	5<br>Agree to a large extent	6<br>Agree strongly	7<br>Totally agree

8. Do you think one should approve according to the Jewish/Islamic religion transplanting a lung taken from a pig in a patient suffering from obstructive pulmonary disease?

1<br>Totally disagree	2<br>Disagree somewhat	3<br>Agree slightly	4<br>Agree moderately	5<br>Agree to a large extent	6<br>Agree strongly	7<br>Totally agree

9. Is it permitted by the Jewish/Islamic religion to do a kidney transplant using a kidney taken from a pig?

1<br>Totally disagree	2<br>Disagree somewhat	3<br>Agree slightly	4<br>Agree moderately	5<br>Agree to a large extent	6<br>Agree strongly	7<br>Totally agree

10. In your opinion, should one approve treatment for a Jewish/Muslim patient who is in danger of death because of pancreatic insufficiency to undergo a transplant of a pancreas/pancreatic cells taken from a pig?

1<br>Totally disagree	2<br>Disagree somewhat	3<br>Agree slightly	4<br>Agree moderately	5<br>Agree to a large extent	6<br>Agree strongly	7<br>Totally agree

11. In your opinion, should one approve a Jewish/Muslim patient to receive an implant of knee cartilage taken from a pig, to replace worn cartilage?

1	2	3	4	5	6	7
Totally disagree	Disagree somewhat	Agree slightly	Agree moderately	Agree to a large extent	Agree strongly	Totally agree

12. In your opinion, should one approve a Jewish/Muslim patient to use skin tissue of a pig for a skin graft after serious burn injuries?

1	2	3	4	5	6	7
Totally disagree	Disagree somewhat	Agree slightly	Agree moderately	Agree to a large extent	Agree strongly	Totally agree
